# A Multiscale Inelastic Internal State Variable Corrosion Model

**DOI:** 10.3390/ma17163995

**Published:** 2024-08-11

**Authors:** M. F. Horstemeyer, W. Song, H. E. Cho, D. Wipf, H. J. Martin, D. K. Francis, S. Chaudhuri

**Affiliations:** 1 School of Engineering, Liberty University, Lynchburg, VA 24515, USA; 2 Department of Mechanical Engineering, Mississippi State University, Starkville, MS 39762, USA; 3 Department of Chemistry, Mississippi State University, Starkville, MS 39762, USA; dwipf@chemistry.msstate.edu; 4 Department of Civil/Environmental and Chemical Engineering, Youngstown State University, Youngstown, OH 44555, USA; hjmartin02@ysu.edu; 5 Center for Advanced Vehicular Systems, Mississippi State University, Starkville, MS 39759, USA; david.francis@leidos.com; 6 Civil, Materials, and Environmental Engineering, University of Illinois at Chicago, Chicago, IL 60607, USA; santc@uic.edu

**Keywords:** corrosion, internal state variable, multiscale, microstructure, damage

## Abstract

We present a corrosion internal state variable (ISV) damage model based upon the integrated computational materials engineering (ICME) hierarchical multiscale paradigm. Structure–property experiments for magnesium alloys were used where the only inputs were the volume fractions of each element of the periodic table. This macroscale ISV corrosion model finds its basis in Horstemeyer’s mechanical damage model, which includes three separate ISVs for damage nucleation, growth, and coalescence, as well as Walton’s inclusion of corrosion, which introduces five new ISVs for pit nucleation, growth, and coalescence, along with general corrosion and intergranular corrosion. While Walton’s corrosion ISVs are phenomenological in nature, herein we develop a multiscale physical basis for the corrosion ISVs. The parameters for the macroscale corrosion ISVs were garnered from the mesoscale Butler–Volmer equations. Pure magnesium with differing amounts of aluminum were used in corrosion tests to exemplify the different pitting, general corrosion, and intergranular corrosion rates, and the macroscale ISV model was calibrated with said data, in which the only inputs to the model are the volume percentages of the elements magnesium and aluminum. Although magnesium alloys were used to motivate and calibrate the model, the model is abstract enough to possibly capture other material systems as well.

## 1. Introduction

The aging of materials in a corrosive environment has become a principal cause for the reduction in the reliability and durability of engineered systems [1,2,3]. Magnesium alloys, as a kind of lightweight material, are receiving increasing attention from automotive and aerospace industries due to their specific high strength-to-weight ratio [4,5]. However, magnesium alloys’ service lives can be significantly reduced when exposed to the external environment because of their high corrosion rate [6]. Hence, the development of an accurate constitutive model that accounts for damage progression effects from mechanical loading and corrosion is required if a simulation-based design considering corrosion is to be used.

An internal state variable (ISV) model is a constitutive theory that can capture the history effect of materials [7,8] and is widely used in continuum damage mechanics [for review, see Horstemeyer and Bammann (2010) [9]]. Coleman and Curtin (1967) [10] laid out the framework of ISV theory, where they used the Clausius–Duhem inequality to place restrictions on the equations’ response functions. Later, Gurson (1977) [11] developed a damage evolution and plasticity model that was essentially an ISV model, although not specifically designated as such. Bammann et al. (1993) [12] employed the Cocks and Ashby (1980) [13] creep void growth rule in an ISV formulism to account for the large deformation kinematics. Horstemeyer et al. (1999, 2000) [14,15] enriched the ISV damage model by including the microstructure features and breaking the damage progression down into separate void nucleation, growth, and coalescence terms. In recent decades, ISV theory has made a big achievement in capturing the damage evolution for materials subjected to mechanical loadings. The ISV damage model and its thermodynamic/kinematic basis has been further expanded by incorporating the effects of hydrogen embrittlement [16], shear damage [17], nuclear irradiation [18], and electromagnetism [19,20].

The corrosion process can also induce damage, reduce material strength, enhance the inelastic flow, and soften the elastic moduli, but ISV theory has not been used to capture damage from corrosion. Walton et al. (2014) [21] developed the first corrosion model based upon ISV theory by using an extended multiplicative decomposition of the deformation gradient. Walton’s ISV corrosion model has the capability to capture macroscale damage, such as general corrosion, pit nucleation, pit growth, pit coalescence, and intergranular corrosion. Later, Amiri et al. (2015) [22] employed an ISV model similar to Walton et al. (2014) [21] but applied it to an aluminum alloy. Chen and Bobaru (2015) [23] proposed a pitting corrosion model to capture the subsurface corrosion damage of metals undergoing an anodic reaction by applying non-local continuum damage (peridynamics) modeling. This model was extended to include a stress-induced corrosion effect [24]. Xia et al. (2022) [25] conducted a literature review of using peridynamics for corrosion modeling. Some researchers employed phase field theory to simulate corrosion [26,27,28]. Hu et al. (2016) [29] applied a damage mechanics approach to account for elastoplastic damage behavior, but for the damage part, they employed Lemaitre and Chaboche’s fatigue damage model [30]. The mechanical damage and pit growth are calculated separately, and the stress state at the center of the pit, which is affected by the geometry of the pit and the fatigue damage in the finite element domain, is estimated with the fatigue damage and pit growth equations. This approach indirectly relates the corrosion effect to their elastoplastic damage constitutive equation. This model was extended to include the thermal damage effect on the thermal corrosion fatigue problem [31] of aluminum alloys. Li et al. (2019) [32] developed a corrosion damage model to account for corrosion initiation and growth kinetics using electrochemical and statistical methods, but without connecting them to mechanical damage. Although several others introduced corrosion models (e.g., [33,34,35]), they are either not cast as ISVs or do not relate to magnesium. Liu and Kelly (2019) [36] conducted a review of the different finite element analyses of localized corrosion effects. Extended finite element methods (XFEMs) were also used to incorporate corrosion fairly recently [37,38,39]. One final numerical method of analyzing corrosion was through cellular automata [40,41,42].

It is commonly known that corrosion is highly dependent on the alloying elements and microstructural features. During the manufacturing process, many of the grain boundaries and grain orientations may be modified. Some impurity elements/second phases may segregate from the matrix, thereby modifying the corrosion stability of the material. However, there is no clear knowledge on how doping and material engineering can be favorably used for altering corrosion rates in promising Mg alloys. A multiscale modeling technique provides a powerful approach to accurately model the corrosion effects by considering the doping effects and the material *microstructure–property* relationships. The aim of this paper was to present a continuum ISV damage corrosion model based upon lower-length-scale analyses so that future applications can use this model in a finite element, peridynamic, phase field, or XFEM environment.

**Figure 1:**
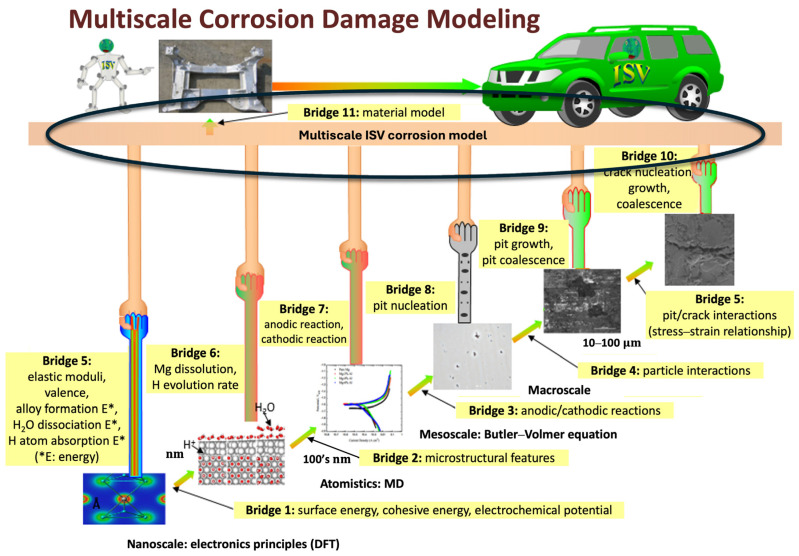
Multiscale schematic of a metal with the consideration of corrosion as the environmental consideration. Note the eleven different bridges of information. In this study, the internal state variable (ISV) model at the macroscale was the main focus, with bridges 1, 5, and 7 informing the ISV model.

In the following sections, a multiscale ISV damage corrosion model is proposed based upon multiscale heterogeneous structures that arise from different elements of the periodic table based on the notion of integrated computational materials engineering (ICME) [43,44]. Figure 1 illustrates the different length scales of importance and the associated information bridges. The ISV model adds the corrosion ISVs to the Bammann et al. (1993) [12] and Horstemeyer et al. (1999; 2000) [14,15] thermomechanical plasticity–damage model, but also relates different corrosion mechanisms (general corrosion, pitting corrosion, intergranular/filiform corrosion) to materials’ microstructural features based on the corrosion framework of Walton et al. (2014) [21]. While the kinematics and thermodynamics are presented in Walton et al. (2014) [21], the multiscale kinetics and corrosion constitutive model are presented herein. Since the reason behind the corrosion damage is anodic and cathodic reactions that occur on material surfaces, the Butler–Volmer equations [45,46,47,48] are applied to bridge the macroscale corrosion damage to the mesoscale electrochemical kinetics (bridge 11 in Figure 1). In turn, mesoscale electrochemical kinetics behaviors can be predicted by including microstructures and nanoscale electrochemical activation energies into the Butler–Volmer equations (bridge 5 and bridge 1, respectively, in Figure 1). As per Horstemeyer (2012) [43], downscaling is required first to determine the information required in the macroscale ISV corrosion damage equations; then, the upscaling occurs, wherein one determines the lower-length-scale equations, parameters, and constants to help feed their results up into the macroscale equations.

Gurtin notation [49] is used to express tensor quantities and their associated mathematical operations. Tensors are denoted by boldface font and scalar values have the standard weights. Uppercase terms (A) represent second-rank tensors and lowercase terms (a) represent first-rank tensors (vectors). The following tensor operations are used in the text: $AB\Rightarrow {\left(A\cdot B\right)}_{ij}=A_{ik}B_{kj},$ $A:B=A_{ij}B_{ij},$ $tr(A)=A_{ii},$ ${\left(A^{T}\right)}_{ij}=A_{ji},$ and $a⊗b={\left(a⊗b\right)}_{ij}$. Special care is given to specify different intermediate configurations by using accent marks, such as tilde $\left(\tilde{B}\right)$, circumflex $\left(\hat{B}\right)$, and macron $\left(\bar{B}\right)$.

## 2. Process–Structure–Property Experiments Used to Calibrate the Corrosion Model

### 2.1. Materials Processing of Magnesium Castings

The Mg plates used herein were tilt pour cast with different percentages (2% and 6%) of aluminum in a ceramic die in order to minimize defects and inclusions. Specimens for immersion corrosion were used similar to that of Martin [50,51,52].

### 2.2. Microstructural Analysis

**Figure 2:**
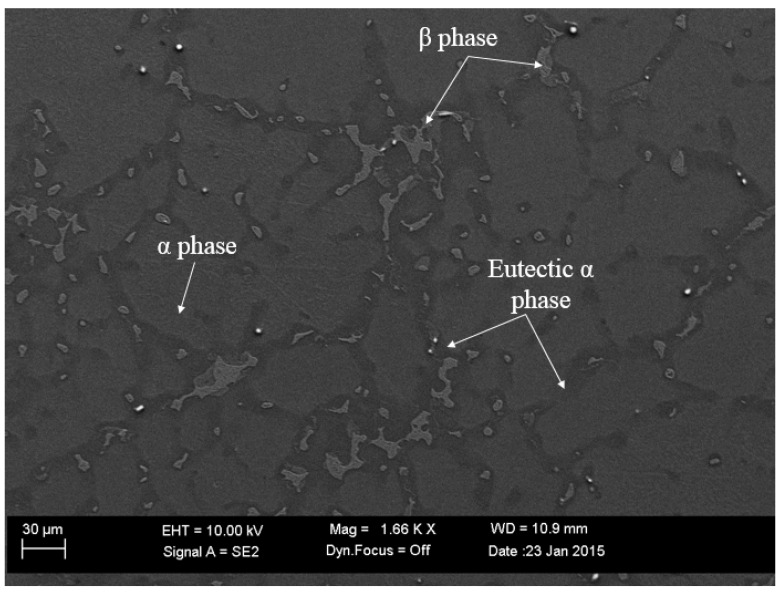
The distribution of the α, eutectic α, and β phases in the Mg-6Al alloy.

For multiphase Mg alloys, the macroscale corrosion damage is affected by three important factors: (i) the chemical composition of each phase, (ii) the fraction of each phase in the material, and (iii) the microstructural distribution of second phases in the material. For the cast Mg-Al alloys, the eutectic α phase usually formed along dendritic arms or grain boundaries and contained a higher level of aluminum in the solid solution. The β phase usually precipitates within the eutectic α phase; with increasing amounts of aluminum in the material, the β phase precipitated more continuously along the grain boundaries and formed a finer lamellar arrangement. Figure 2 shows the distribution of the α, eutectic α, and β phases in the Mg-6Al magnesium alloy. As the eutectic α phase and β phase had lower corrosion rates than the α phase, they could improve the overall corrosion resistance of the material. On the other hand, because the eutectic α phase and β phase had more positive electrochemical potential, they could act as effective cathodic sites for the α phase matrix and accelerate the dissolution rate of the α phase [53]. Therefore, in order to capture the corrosion behavior of Mg alloys accurately, we needed to consider both the corrosion resistance improvement and anodic dissolution acceleration affected by the second phases.

Liu et al. (2009) [53] quantified the eutectic α phase and β phase (Mg_17_Al_12_) for Mg-Al alloys ranging from 0.5 wt% Al to 12 wt% Al and found that the volume fraction and the sizes of both phases increased with the addition of aluminum content and followed an almost linear relationship. However, due to different parameters in the casting process, such as the cooling rate and heat treatment, the microstructure for the same Mg alloys was discovered to be different. 

Table 1 summarizes the pertinent microstructural data. GS(ξ) is the grain size (μm), $D_{Eu\_\alpha }(\xi )$ is the particle size/phase size (μm) of the eutectic α phase, $\delta_{Eu\_\alpha }(\xi )$ is the number density (mm^−2^) of the eutectic α phase, $NND_{Eu\_\alpha }(\xi )$ is the nearest neighbor distance (μm) of the eutectic α phase, and $f_{Eu\_\alpha }(\xi )$ is the area fraction of the eutectic α phase in the material. $P_{matrix}(\xi )$ is the aluminum content (wt.%) in the eutectic α phase, and $P_{matrix}$ is the aluminum content (wt.%) in the matrix material. $D_{\beta }(\xi )$, $\delta_{\beta }(\xi )$, $NND_{\beta }(\xi )$, and $f_{\beta }(\xi )$ are the particle size (μm) of the β phase, particle number density (mm^−2^) of the β phase, nearest neighbor distance (μm) between β phase particles, and area fraction of the β phase.

### 2.3. Corrosion Properties

Mg-Al specimens (2.54 cm × 2.54 cm × varying thicknesses) were cut from the cast plates. Each specimen surface with its particular chemical composition was left untreated and directly placed into the saltwater bath. For immersion testing, an aquarium with an aeration unit was filled with 3.5 wt.% NaCl at room temperature. For both tests, the six specimens per test environment were hung at 20° to the horizontal, as recommended by ASTM B-117 [54]. Measurements of the weight change, number density of pits, pit size (volume), nearest neighbor distance of pits, and intergranular area fraction were made at each time increment. The specimens were exposed to the test environment for 1 h, removed, rinsed with distilled water to remove excess salt, and dried. The specimens were then placed back into the test environment for an additional 3 h, an additional 8 h, an additional 24 h, and another 24 h. These times allowed for a longitudinal study to follow the pit growth and surface changes over time, where *t*_0_ = 0, *t*_1_ = 1 h, *t*_2_ = 4 h, *t*_3_ = 12 h, *t*_4_ = 36 h, and *t*_5_ = 60 h. Between the measurements and environmental exposures, the specimens were stored in a desiccator to ensure that no further surface reactions occurred. The data are shown later with the calibration of the ISV corrosion damage model with their associated error bands.

**Figure 3:**
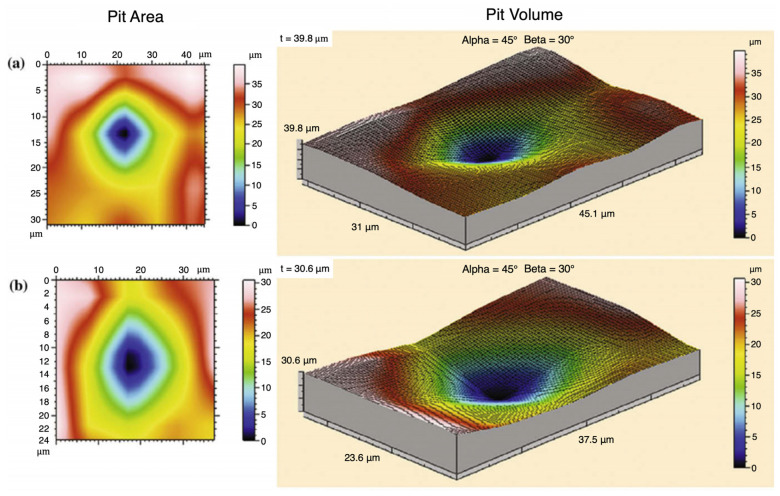
Laser profilometry that measured the pit depth and width to determine the volume of a magnesium specimen with six percent aluminum. Two pits are shown ((**a**) top and (**b**) bottom). The color contours show the area in the left hand column and the volume in the right hand column.

After each increment of testing, the immersion specimens were analyzed with a laser profilometer (Talysurf CLI 2000, Taylor Hobson Precision Ltd., Leicester, UK) before being placed back into the aquarium for additional corrosion testing. Figure 3 illustrates a picture of a corrosion pit measured by the laser profilometer for the magnesium specimen with 6% Al. Laser profilometry was performed following each corrosion increment on the front face of each immersion specimen. A 1 mm × 1 mm area was scanned using the laser beam for a duration of 3 h 42 min. The scanning speed was 500 µm/s, with a spacing of 0.5 µm and a resolution of 2001 points. Upon completion of the corrosion testing, the laser profilometry 2D and 3D images were visually analyzed for signs of pitting.

Following the 1500 h of corrosion testing, Zeiss SUPRA 40 field emission gun scanning electron microscopy (FEG-SEM) was performed on the face of one of the immersion samples at several locations. Energy-dispersive X-ray spectroscopy (EDS) was performed on the corrosion product formed during the tests to determine the exact chemical composition of the precipitates. After all the testing was complete, a Struers diamond saw (Struers, Cleveland, OH, USA) with a diamond-coated copper blade was used to bisect one of the salt fog samples for further analysis. The bisected sample was analyzed with SEM, followed by X-ray mapping of the SEM images to determine whether or not pitting was present.

Other measurements were made with the specimens regarding the electrochemical potentials in terms of their polarization resistance (R_p_). Table 2 summarizes the results for pure Mg and the alloys with different amounts of aluminum. For example, *E_alpha__*_1_._5_*_Al_* designates that 1.5% aluminum is within the alpha magnesium and so on. *E_β_* is the potential for the beta phase of magnesium. All electrochemical experiments were executed in a conventional undivided three-electrode cell using a saturated calomel electrode (SCE) as the reference electrode and the Mg-Al specimen as the working electrode. The resistance polarization and corrosion potential values were measured in 3.5 wt. % NaCl solution using a Solartron Analytical 1470E potentiostat/galvanostat (Ametek Scientific Instruments, Oak Ridge, TN, USA). Corrosion potentials (*E_corr_*) and resistance polarization values were determined by fitting polarization curves using the CView program (Scribner Associates).

## 3. Macroscale Corrosion Damage

The damage parameter ϕ, which was described by Walton et al. (2014) [21], was updated to capture the corrosion damage evolution. The total corrosion damage can be defined as

(1) $$\phi_{total}=\phi_{gc}+\phi_{pc}+\phi_{ic}$$

where $\phi_{gc}$ is the general corrosion damage, $\phi_{pc}$ is the localized pitting corrosion damage, and $\phi_{ic}$ is the intergranular corrosion damage. The parameters that affect the three distinct terms in Equation (1) that are NOT in Walton et al. (2014) [21] are the following: (i) the type and stereology of the second-phase particles, (ii) base materials’ chemical composition, and (iii) microstructural features. In addition, filiform corrosion may substitute for intergranular corrosion. The associated damage rate equation is obtained in the following form:

(2) $${\dot{\phi }}_{total}={\dot{\phi }}_{gc}+{\dot{\phi }}_{pc}+{\dot{\phi }}_{ic}$$

Walton et al. (2014) [21] developed an equation to account for the relative pitting corrosion damage:

(3) $$\phi_{pc}=\eta_{p}v_{p}c_{p}$$

where $\eta_{p}$ represents the pit nucleation related to the pit number density; $v_{p}$ represents the pit growth related to the pit in-plane area, pit depth, and pit volume; and $c_{p}$ is the pit coalescence term related to the nearest neighbor distance between pits.

### 3.1. Macroscale General Corrosion Rate

General corrosion can be referred to as corrosion that attacks an exposed surface uniformly without inducing appreciable localization. General corrosion can be obtained by measuring the mass change or thickness change of a specimen. Faraday’s law [55,56] proposes that the mass loss of an electrode during electrolysis is directly proportional to the quantity of electricity transferred at that electrode:

(4) $$m=\frac{QM}{Fz}$$

where m is the mass change of an electrode in grams; Q is the total electric charge passed through the substance; F is the Faraday constant; M is the molar mass of the substance; and z is the valency number of ions of the substance, where z=2 for magnesium. For the general corrosion rate ${\dot{\phi }}_{gc}$, a modified Faraday’s law is proposed in an ISV form:

(5) $${\dot{\phi }}_{gc}=C_{1}(C_{2}+\phi_{gc})\frac{M}{Fz}$$

where $C_{1}$ and $C_{2}$ are material parameters that are determined by the material properties related to *Q*, which is the electric charge.

### 3.2. Macroscale Pitting Corrosion

Pitting corrosion is a form of extremely localized corrosion that results in the creation of small holes in the metal. Pitting corrosion is usually defined as an autocatalytic process [57]. The spatial separation of the cathodic and anodic half-reactions generates a potential gradient and promotes the electromigration of aggressive anions into the pits, causing pit nucleation, growth, and coalescence. Pits are susceptible to nucleate at impurities, inclusions, grain boundaries, or surface defect/flaw regions because these inhomogeneous sites can facilitate the breakdown of the passive films [58]. The pit nucleation rate can be written in the ISV form as

(6) $$\dot{\eta }=\left\{ \begin{array}{l} C_{3}(C_{4}-\eta )\;\mathrm{if}\;t<t_{c}\\ C_{5}(C_{6}-\eta )\;\mathrm{if}\;t\geq t_{c} \end{array}\right.$$

where $C_{3}$ is the rate of nucleation pitting, $C_{4}$ is the saturation rate of the accelerating pit number density, $C_{5}$ is the decelerating rate of pitting as coalescence takes over, and $C_{6}$ is the saturation rate of the deceleration. These parameters (*C*_3_–*C*_6_) are functions of the second phase/particle volume fraction, particle size, temperature, and pH level. $t_{c}$ is the transition time from pit-nucleation-dominated to pit-coalescence- or general-corrosion-dominated behavior.

The pit growth rate and stability of the active corrosion are dependent on the material composition, aggressive electrolyte concentration, formation and dissolution of the corrosion film, and the potential inside the pit [59]. The pit growth equation is expressed in the following form:

(7) $${\dot{v}}_{p}=\left\{ \begin{array}{l} C_{7}(C_{8}+\nu )\;\mathrm{if}t<t_{c}\\ C_{9}(C_{10}+\nu )\;\mathrm{if}\;t\geq t_{c} \end{array}\right.$$

where $C_{7}$ is the rate of velocity of the pit growth, *C*_8_ is the saturation of the pit growth velocity, *C*_9_ is the decelerating rate of the pit growth as the coalescence takes over, and *C*_10_ is the saturation pit growth rate of the deceleration. These parameters (*C*_7_–*C*_10_) are functions of the second phase/particle volume fraction, particle size, temperature, and pH level. v is the average pit volume on the specimen surfaces; please note that v can also be the average in-plane pit area if you measure the pit area instead of the pit volume.

In practice, pits will interact with neighboring pits and coalesce into larger pits. The coalescence rate $\dot{C}$ is assumed to relate to the electrochemical interaction forces between pits. Walton et al. (2014) [21] proposed a pit coalescence model based on Coulomb’s Law [60,61,62] and Maxwell’s stress [63]. The original form of Coulomb’s Law is

(8) $$F_{c}=\frac{k_{e}q_{1}q_{2}}{r^{2}}$$

where $F_{c}$ is the electrostatic interaction force between pits, $k_{e}$ is the Coulomb constant ($k_{e}=8.987\times 10^{9}\;{\mathrm{Nm}}^{2}/C^{2}$), and $q_{1}$ and $q_{2}$ are point charges. r is the separation distance between two pits, which is defined as the nearest neighbor distance (NND) of the corrosion pits. By combining with both Coulomb’s Law and Maxwell stress together, we arrived at the following pitting coalescence rate in an ISV form as the following:

(9) $${\dot{c}}_{p}=\frac{k_{e}q_{1}q_{2}}{\varepsilon_{0}\pi {\left(N\dot{N}D(t)\right)}^{4}}$$

(10) $$N\dot{N}D=\left\{ \begin{array}{l} C_{11}(C_{12}-NND)\;if\;t<t_{c}\;\\ C_{13}(C_{14}-NND)\;if\;t\geq t_{c} \end{array}\right.$$

where $\varepsilon_{0}$ is the electric constant and equals 8.854 × 10^−12^ F/m. *C*_11_ is the rate of pitting coalescence, *C*_12_ is the saturation of the pit coalescence, *C*_13_ is the decelerating rate of the pit coalescence, and *C*_14_ is the saturation pit coalescence of the deceleration. These parameters (*C*_11_–*C*_14_) are functions of the second phase/particle volume fraction, particle size, temperature, and pH level.

### 3.3. Macroscale Intergranular Corrosion

Intergranular corrosion is a localized attack along grain boundaries or regions adjacent to the grain boundaries [64]. This form of corrosion is mainly induced by the uneven distribution of the chemical composition because impurities or second phases tend to form on the grain boundaries; in essence, the segregation of the chemical composition induces galvanic cells along grain boundaries. The intergranular corrosion rate $\phi_{ic}$ is formulated in the following ISV form:

(11) $${\dot{\phi }}_{ic}=\left\{ \begin{array}{l} C_{15}(C_{16}-\phi_{ic}){\left(\frac{MO}{MO_{0}}\right)}^{z_{ic}}\;\mathrm{if}\;t<t_{c}\\ C_{17}(C_{18}+\phi_{ic}){\left(\frac{MO}{MO_{0}}\right)}^{z_{ic}}\;\mathrm{if}\;t\geq t_{c} \end{array}\right.$$

where *C*_15_ the rate of intergranular corrosion, *C*_16_ is the saturation of the intergranular corrosion, *C*_17_ is the decelerating rate of the intergranular corrosion, and *C*_18_ is the saturation intergranular corrosion of the deceleration. These parameters (*C*_15_–*C*_18_) are functions of the second phase/particle volume fraction, particle size, temperature, and pH level. ${\left(\frac{MO}{MO_{0}}\right)}^{z_{ic}}$ is a grain misorientation factor. The misorientation factor describes the distribution of the crystallographic orientations in adjacent grains and can be obtained from the EBSD/pole figure results. Birbilis et al. (2010) [65] is a good example of a study that showed a direct dependence on the grain features and corrosion for a magnesium alloy.

### 3.4. Mesoscale Electrochemical Kinetics

The Butler–Volmer equations [45,46,47,48] describe the kinetics for electrochemical reactions, which are controlled by the transfer of charge across the interfaces. The Butler–Volmer equations link four very important parameters together: the Faradaic current, the electrode potential, the concentration of reactants, and the concentration of the products.

(12) $$i=i_{corr}\cdot \left\{\exp\left[\frac{\alpha_{a}nF_{R}}{RT}\Delta E\right]-\exp\left[-\frac{\alpha_{c}nF_{R}}{RT}\Delta E\right]\right\}$$

(13) $$\Delta E=(E-E_{eq})$$

In Equation (12), the first term represents the anodic partial current density, while the second term is the cathodic partial current density, where i is the electrode current density, $i_{corr}$ is the exchange current density, ΔE is the overpotential, E is the electrode potential, and $E_{eq}$ is the equilibrium/reversible potential. From the above relationships, the net current (i) is positive when the electrode is anodically polarized and negative when the electrode is cathodically polarized. n is the number of electrons involved in the electron reaction; $F_{R}$ is the Faraday constant; R is the universal gas constant; and $\alpha_{a}$ and $\alpha_{c}$ are the so-called anodic and cathodic charge transfer coefficients, respectively. Note that the values of $\alpha_{a}$ and $\alpha_{c}$ do not necessarily sum to unity but are related by

(14) $$\alpha_{a}+\alpha_{c}=\frac{1}{\upsilon }$$

where υ is the stoichiometric number, or the number of times that the rate-determining step must occur for the overall reaction to occur once.

When the anodic polarization potential is significantly larger than the equilibrium potential (*η_α_* > 50 mV), the first term of the Equation (12) dominates the second term. Thus, the Butler–Volmer equation can be simplified to

(15) $$i=i_{0}\exp\left[\frac{\alpha_{a}nF_{R}\Delta E}{RT}\right]$$

Rearranging Equation (15), one obtains the Tafel equation [66,67]:

(16) $$\eta_{a}=\beta_{a}\log\left(\frac{i}{i_{0}}\right)$$

where $\beta_{a}$ is the anodic Tafel slope, and $\beta_{a}$ can be associated with $\alpha_{a}$ in the following form:

(17) $$\beta_{a}=\frac{2.3RT}{\alpha_{a}nF_{R}}$$

A similar equation is obtained for the cathodic activation polarization:

(18) $$\eta_{c}=-\beta_{c}\log\left(\frac{\left|i\right|}{i_{0}}\right)$$

where $\beta_{c}$ is the cathodic Tafel slope, and it relates to $\alpha_{c}$ by

(19) $$\beta_{c}=\frac{2.3RT}{\alpha_{c}nF_{R}}$$

According to the Stern–Geary equation (1957) [68], the exchange corrosion current can be determined by a small polarization from the corrosion potential:

(20) $$i_{corr}=\frac{\beta_{a}\beta_{c}}{(\beta_{a}+\beta_{c})2.3R_{p}}$$

where $R_{p}$ is the polarization resistance, which can be defined as the slope of the linear polarization curve at the corrosion potential. For our model, we regard $R_{p}$ as a material property, and it does not change over the exposure time; note that the units of $R_{p}$ are $\Omega \;{\mathrm{cm}}^{-2}$. When one substitutes Equations (17) and (19) into Equation (20), one obtains

(21) $$i_{corr}=\frac{RT}{nF(\alpha_{a}+\alpha_{c})R_{p}}$$

When substituting the exchange current Equation (21) into the Butler–Volmer equation (Equation (15)), the corrosion current can be changed to the following form:

(22) $$i=\frac{RT}{nF(\alpha_{a}+\alpha_{c})R_{p}}\exp\left[\frac{\alpha_{a}nF_{R}\Delta E}{RT}\right]$$

The corrosion current density i that is determined from the electrochemical kinetic part can be used to quantify the corrosion rate that occurs on the surface. The rate of material loss r (g h^−1^) is associated with the corrosion current density i (A cm^−2^) by

(23) $$r=i\frac{M}{zF}\;\mathrm{from}\;m=\frac{QM}{zF}$$

The corrosion current i is the sum of the general corrosion current $i_{gc}$ and the pitting corrosion current $i_{pc}$ together. Equation (23) comes from Faraday’s law of electrolysis [55,56], where m is the mass loss and Q is the electrical charge given by Q=∫i dt. Hence, we now can relate the resistance *R_p_* with the corrosion damage (Equation (5)).

### 3.5. Bridging Different Length Scales

The different length scales in the corrosion damage model are represented by different microstructures at a variety of length scales. Measurements of the microstructures were evaluated by their weight percentage ξ, and different equations were developed by trial and error to give the following relationships:

(24) $$GS(\xi )=370599\xi^{2}-37012\xi +1025.8$$

$$D_{Eu\;\alpha }(\xi )=358.32\xi +1.0318,\;\delta_{Eu\_\alpha }(\xi )=6903\xi -7.6459$$

$$NND_{Eu\_\alpha }(\xi )=114.25\xi +38.285,\;f_{Eu\_\alpha }(\xi )=-3.407\xi +0.4044$$

$$P_{Eu\_\alpha }(\xi )=25.012\xi^{2}+0.0993\xi +0.002,\;P_{matrix}(\xi )=0.5007\xi$$

$$D_{\beta }(\xi )=1944\xi^{2}-20.223-0.0921$$

$$\delta_{\beta }(\xi )=41518\xi^{2}+446.9\xi -443.926$$

$$NND_{\beta }(\xi )=-436.13\xi +39.707,\;f_{\beta }(\xi )=(7.3\times 10^{-6}e^{145.3\xi })$$

E(ξ)=1.6667ξ−1.663

where GS(ξ) is the grain size (μm), $D_{Eu\_\alpha }(\xi )$ is the particle size/phase size (μm) of the eutectic α phase, $\delta_{Eu\_\alpha }(\xi )$ is the number density (mm^−2^) of the eutectic α phase, $NND_{Eu\_\alpha }(\xi )$ is the nearest neighbor distance (μm) of the eutectic α phase, and $f_{Eu\_\alpha }(\xi )$ is the area fraction of the eutectic α phase in the material. $P_{matrix}(\xi )$ is the aluminum content (wt.%) in the eutectic α phase and $P_{matrix}$ is the aluminum content (wt.%) in the matrix material. $D_{\beta }(\xi )$, $\delta_{\beta }(\xi )$, $NND_{\beta }(\xi )$, and $f_{\beta }(\xi )$ are the particle size (μm) of the β phase, particle number density (mm^−2^) of the β phase, nearest neighbor distance (μm) between the β-phase particles, and the area fraction of the β phase, respectively. E(ξ) is the electrochemical potential value of the Mg-ξ Al solution phase.

#### 3.5.1. General Corrosion Rate at the Mesoscale

General corrosion removes the material surface uniformly, and its rate is affected by the chemical composition of each phase and the area fraction of each phase in the material. The general corrosion rate can be changed over time due to a corrosion film that forms on the surface, thus increasing the surface roughness [66]. For instance, when one places a fresh magnesium specimen into 3.5% NaCl solution, the corrosion current can be higher than the condition when a corrosion film uniformly covers the specimen surface. In addition, with the increase in corrosion time, localized corrosion pits will be generated, leading to the breakdown of the corrosion film, thus further increasing the surface roughness. Then, the general corrosion rate can be much greater as time progresses. We note that Table 3 and Table 4 share the values for each constant that is described in the following subsections. We chose model constants set with the lowest error between the model curve and the experimental data by performing a least-squares optimization. Note that the calibrated model constants and used parameters are summarized in Table 3 and Table 4. 

Related to the modified Butler–Volmer equations, the corrosion resistance can be improved by adding in the effect of the second phases as follows:

(25) $$R_{p}(\xi )=\frac{R_{p}}{\left[1-20.6\;P_{matrix}(\xi )f_{matrix}(\xi )+15.3\;P_{eu\_\alpha }(\xi )f_{eu\_\alpha }(\xi )-15.5\;P_{\beta }(\xi )f_{\beta }(\xi )\right]}$$

where $R_{p}(\xi )$ is the overall polarization resistance of the Mg-ξ Al alloy and $R_{p}$ is the polarization resistance of the pure Mg.

As the dissolution rate of the α phase can be affected by the second phases, the general corrosion current can be written as the following:

(26) $$\begin{array}{l} i_{gc}(\xi )=\frac{1.58RT}{nF_{R}(\alpha_{a}+\alpha_{c})R_{p}(\xi )}\cdot \frac{k_{1}}{g(\xi )}\cdot \exp[\frac{\alpha_{a}\left(\xi \right)nF_{R}}{RT}\cdot \\ \;\;\;\left(\left(E_{Eu\_\alpha }(\xi )-E_{\alpha }(\xi )\right)f_{Eu\_\alpha }(\xi )+\left(\left(E_{\beta }(\xi )-E_{\alpha }(\xi )\right)f_{\beta }(\xi )\right)\right)] \end{array}$$

where $k_{1}$ is a parameter that can be used to adjust the unit of the current density; if the unit of general current is ($A/(m^{2}\cdot h)$, $k_{1}$ equals 3600. The value of ($\alpha_{a}+\alpha_{c}$) is assumed to be 0.5, $\alpha_{a}(\xi )$ is the anodic transfer coefficient of the Mg-ξ Al alloy, and g(ξ) is a coefficient that indicates how the positive potential of the second phases affects the corrosion rate, where

(27) $$\alpha_{a}(\xi )=2.6043\xi +0.3264$$

(28) $$g(\xi )=5247.9\xi^{2}-720.17\xi +24.17$$

When considering the general corrosion rate (Equation (5)) with the Butler–Volmer equation and the microstructural features, the general corrosion rate can be formulated with only the percent weight fraction of magnesium and aluminum as follows:

(29) $$\begin{array}{ll} {\dot{\phi }}_{gc}= & \frac{1.58RT\cdot \left[1-20.589P_{matrix}(\xi )f_{matrix}(\xi )+15.261P_{eu\_\alpha }(\xi )f_{eu\_\alpha }(\xi )-15.527P_{\beta }(\xi )f_{\beta }(\xi )\right]}{nF_{R}(\alpha_{a}+\alpha_{c})}\;\cdot \\ & \left\{\frac{k_{1}}{g(\xi )R_{p}}\exp\left[\frac{\alpha_{a}\left(\xi \right)nF_{R}}{RT}\left(\left(E_{Eu\_\alpha }(\xi )-E_{\alpha }(\xi )\right)f_{Eu\_\alpha }(\xi )+\left(\left(E_{\beta }(\xi )-E_{\alpha }(\xi )\right)f_{\beta }(\xi )\right)\right)\right]+\phi_{gc}\right\}\;\cdot \;\frac{M}{nF_{R}} \end{array}$$

#### 3.5.2. Pit Nucleation Rate

When corrosion specimens have the same initial roughness and are tested in the same corrosive environment, the pit nucleation rate depends on the material’s corrosion resistance and particle number density. The pit number density usually increases with exposure time to a certain level, after which, the pit number decreases due to the influences of pit coalescence and general corrosion. It is commonly known that a pit tends to nucleate from particles/second phases; for Mg-Al alloys, pits tend to nucleate around the eutectic α phase and β phase. However, the β phase is formed within the eutectic α phase. With the increase in aluminum content in the material, more eutectic α phase will change into the β phase. Thus, for Mg-Al alloys, only the dominated phase’s particle number density needs to be considered to predict the pit nucleation rate. Before the pit number density (mm^−2^) reaches the critical point (${\dot{\eta }}_{\xi }\approx 0$), the pit nucleation rate can be formulated as

(30) $$\dot{\eta }(\xi )=\frac{2.5\cdot h_{1}(\xi )}{\left(R_{p}(\xi )/R_{p}\right)}\cdot \left[\left(1650+C_{nucleation\;1}\cdot \langle \delta_{eu\_\alpha }\left(\xi \right),\delta_{\beta }\left(\xi \right)\rangle )-\eta (\xi \right)\right]$$

(31) $$h_{1}(\xi )=-1934\xi^{3}+216.5\xi^{2}-74.9\xi +1$$

(32) $$C_{nucleation1}=\left(1.25,0.523,2.015\right)$$

When comparing the ISV pit nucleation rate from Equation (6) and microstructure-dependent Equations (30)–(32), one can see that *C*_3_ = 2.5*h*_1_(*ξ*)*/*[R*_p_*(*ξ*)*/*R*_p_*)] and *C_4_* = (1650 + *C_nucleation_*_1_$\langle \delta_{eu\_\alpha },\delta_{\beta }\rangle$). Here, $h_{1}(\xi )$ is a calibration parameter that is used to account for how the microstructure-dependent corrosion resistance affects the pit nucleation rate. $C_{nucleation\;1}$ is the coefficient for the pit nucleation, where $C_{nucleation\;1}$ equals 1.25 when the particle number density of the eutectic phase is greater; otherwise, $C_{nucleation\;1}$ equals 0.523 for pure magnesium. However, there is an exceptional case: if $\delta_{\beta }\geq \delta_{Eu\_\alpha }$ and the β phase is surrounded by a small region of pure Mg, $C_{nucleation\;1}$ will equal 2.015 because the corrosion pit will nucleate more easily. $\langle \delta_{eu\_\alpha },\delta_{\beta }\rangle$ is a term that indicates the maximum value between $\delta_{eu\_\alpha }$ and $\delta_{\beta }$.

After the pit nucleation reaches the saturation level, the pit nucleation rate can be modeled in the following form:

(33) $$\dot{\eta }(\xi )=\frac{2.5\cdot h_{2}(\xi )}{\left(R_{p}(\xi )/R_{p}\right)}\cdot \left[\left(130+C_{nucleation\;2}\cdot \langle \delta_{eu\_\alpha }\left(\xi \right),\delta_{\beta }\left(\xi \right)\rangle )-\eta (\xi \right)\right]$$

(34) $$h_{2}(\xi )=1+2.07e^{-\frac{{\left(\xi -0.04\right)}^{2}}{0.00018}}$$

(35) $$C_{nucleation\;2}=(5.58,0.60669,2.4317)$$

When comparing the ISV pit nucleation rate from Equation (6) in the deceleration side of the equation and microstructure-dependent Equations (33)–(35), one can see that *C*_5_ = 2.5*h*_2_(*ξ*)*/*[R*_p_*(*ξ*)*/*R*_p_)*] and *C_6_* = (130 + *C_nucleation_*_2_$\langle \delta_{eu\_\alpha },\delta_{\beta }\rangle$). $C_{nucleation\;2}$ equals 5.58 when the particle number density of the eutectic phase is greater; otherwise, $C_{nucleation\;2}$ equals 0.60669 in pure magnesium. If $\delta_{\beta }\geq \delta_{Eu\_\alpha }$ and the β phase is surrounded by a small region of pure Mg, $C_{nucleation\;2}$ equals 2.4317.

#### 3.5.3. Internal State Variable (ISV) Pit Growth Rate with Butler–Volmer Equation

With a negative corrosion potential, Mg and its alloys are prone to be subject to microgalvanic corrosion when there are potential differences between the primary α, eutectic α, and β phases [69]. Unlike general corrosion, galvanic pitting corrosion is more localized, as it is affected by the matrix material’s corrosion resistance, the relative electrochemical difference between the second phase and matrix material, and the protection performance of the corrosion film formed on the surface. The Butler–Volmer equation is a kind of mesoscale model; it is generally used to predict how the current flow goes through a certain surface area; however, it cannot directly be used to predict the pit growth rate. The pit growth rate was formulated by referring to the current growth rate in the Butler–Volmer equation and expressed as follows:

(36) $${\dot{\nu }}_{pc}(\xi )=\frac{1}{f_{1}(\xi )\cdot (R_{\alpha }(\xi )/R_{p})}\exp\left(\frac{\alpha_{a}nF_{R}}{RT}\Delta E(\xi )\right)\left(1350-\nu_{pc}\right)$$

(37) $$\begin{array}{ll} \Delta E\left(\xi \right)= & E_{\beta }(\xi )-E_{\alpha }(\xi )\cdot (D_{\beta }(\xi )\cdot 10^{-6}/(1/\delta_{\beta }(\xi ))+\\ & \left(E_{Eu\_\alpha }(\xi )-E_{\alpha }(\xi ))\cdot (D_{eu\_\alpha }(\xi )\cdot 10^{-6}/(1/\delta_{eu\_\alpha }(\xi )\right) \end{array}$$

(38) $$f_{1}(\xi )=5687\xi^{2}-50.9\xi +10$$

When comparing the ISV pit growth rate from Equation (7) and the microstructure-dependent Equations (36)–(38), one can see that the rate of change of the pit growth *C*_7_ = exp[*α_a_nF_R_ ΔE*(*ξ*)/(*RT*)]/[*f*_1_(*ξ*)(*R_α_*(*ξ*)/*R_p_*], and the saturation pit growth rate is *C*_8_ = 1350. Here, $f_{1}(\xi )$ is a material parameter that accounts for how the overpotential and polarization resistance affect the pit growth rate. With the increase in exposure time, other corrosion mechanisms may become dominated on the surfaces and affect the pit growth rate. The material parameter will change to another form, i.e., $f_{2}(\xi )$, and the pit growth rate becomes

(39) $${\dot{\nu }}_{pc}(\xi )=\frac{1}{f_{2}(\xi )\cdot (R_{\alpha }(\xi )/R_{p})}\exp\left(\frac{\alpha_{a}nF}{RT}\Delta E(\xi )\right)\left(1350-\nu_{pc}\right)$$

(40) $$f_{2}(\xi )=-6070\xi^{2}+316.82\xi +9.97$$

When comparing the ISV pit growth rate from Equation (7) and the microstructure-dependent Equations (39) and (40), one can see that the rate of change of pit growth *C*_9_ = exp[*α_a_nF_R_ΔE*(*ξ*)/(*RT*)]/[*f*_2_(*ξ*)(*R_α_*(*ξ*)/*R_p_*], and the saturation pit growth rate is *C*_10_ = 1350.

#### 3.5.4. Internal State Variable (ISV) Pit Coalescence Rate with Butler–Volmer Equation

As the pit coalescence rate is assumed to relate to the electrochemical interaction forces between pits, the “pulling force” strength will increase rapidly with the decrease in the nearest neighbor distance between pits. The pit coalescence rate is given as

(41) $${\dot{c}}_{p}=\frac{k_{e}q_{1}q_{2}}{\varepsilon_{0}\pi {\left(N\dot{N}D(t)\right)}^{4}}$$

(42) $$N\dot{N}D(\xi )=\hbar_{1}(\xi )\frac{1}{\left(R_{\alpha }(\xi )/R_{p}\right)}\left(\frac{D_{0}}{d_{NND}}\right)\cdot \exp\left(\frac{\alpha_{a}nF_{R}}{RT}\Delta E(\xi )\right)\cdot (10.2-NND)$$

(43) $$\hbar_{1}(\xi )=0.0131\xi^{0.654}$$

When comparing the ISV pit coalescence rate from Equations (9) and (10) and the microstructure-dependent Equations (41)–(43), one can see that the rate of change of pit coalescence *C*_11_ = $\hbar_{1}\left(\xi \right)$(*D*_0_*/d_NND_*)exp[*α_a_nF_R_ ΔE*(*ξ*)/(*RT*)]/[(*R_α_*(*ξ*)/*R_p_*], and the threshold pit coalescence rate is *C*_12_ = 10.2. Here, $\hbar_{1}\left(\xi \right)$ is a calibration parameter for each material, and the NND equal to 10.2 μm is a threshold value at which the pits coalesce with each other. After the NND reaches this threshold, the nearby pits have already grown into a larger pit, and thus, the pit NND will increase with the exposure time:

(44) $$N\dot{N}D=0.1\frac{1}{\left(R_{\alpha }(\xi )/R_{p}\right)}\left(\frac{{D_{0}}^{2}}{d_{NND}}\right)\cdot \exp\left(\frac{\alpha_{a}nF_{R}}{RT}\Delta E(\xi )\right)\cdot \left(\left(\frac{1200}{\eta_{60}}+10.2\right)-NND\right)$$

where $\eta_{60}$ is the pit number density at 60 h. Then, the rate of change of pit coalescence *C*_11_= 0.1(*D*_0_^2^*/d_NND_*)exp[*α_a_nF_R_ ΔE*(*ξ*)/(*RT*)]/[(*R_α_*(*ξ*)/*R_p_*], and the threshold pit coalescence rate is *C*_12_ = (1200/*η*_60_) + 10.2. 

#### 3.5.5. Internal State Variable (ISV) Intergranular Corrosion Rate with Butler–Volmer Equation

Intergranular corrosion is usually formed on the specimen surface when there are impurity phases along the grain boundaries. The intergranular corrosion rate is affected by the grain size, grain orientation, particle size, and number density along the grain boundary:

(45) $${\dot{\phi }}_{ic}(\xi )=0.3\cdot \left(\kappa_{1}(\xi )\cdot \exp\left[\begin{array}{l} \frac{\alpha_{a}\left(\xi \right)nF_{R}}{RT}[\left(E_{Eu\_\alpha }(\xi )-E_{\alpha }(\xi )\right)f_{Eu\_\alpha }(\xi )\\ +\left(\left(E_{\beta }(\xi )-E_{\alpha }(\xi )\right)f_{\beta }(\xi )\right) \end{array}\right]\cdot GS\left(\xi \right)-\phi_{ig}\right){\left(\frac{MO}{MO_{0}}\right)}^{z_{ic}}$$

(46) $$\kappa_{2}(\xi )=0.0025\xi +0.0003$$

When comparing the ISV pit coalescence rate from Equation (11) and the microstructure-dependent Equations (45) and (46), one can see that the rate of change of intergranular corrosion *C*_15_ = 0.3, and the threshold intergranular corrosion rate is *C*_16_ = *κ*_1_(*ξ*)exp[*α_a_*(*ξ*)*nF_R_*/(*RT*)][(*E_Eu_α_* (*ξ*))*f_Eu_α_* (*ξ*) + (*E_β_* (*ξ*) − *E_a_*(*ξ*))*f_β_* (*ξ*)]*GS*(*ξ*).

With the increase in experiment time, the area fraction of second phase particles along the grain boundary will affect the rate the most, and the intergranular corrosion rate becomes

(47) $${\dot{\phi }}_{ic}(\xi )=0.007\cdot \left(\begin{array}{l} \kappa_{2}(\xi )\cdot \left(f_{Eu\_\alpha }\left(\xi \right)+f_{Eu\_\beta }\left(\xi \right)\right)\cdot \\ \exp\left[\begin{array}{l} \frac{\alpha_{a}\left(\xi \right)nF_{R}}{RT}[\left(E_{Eu\_\alpha }(\xi )-E_{\alpha }(\xi )\right)f_{Eu\_\alpha }(\xi )\\ +\left(\left(E_{\beta }(\xi )-E_{\alpha }(\xi )\right)f_{\beta }(\xi )\right) \end{array}\right]\cdot GS\left(\xi \right)+\phi_{ig} \end{array}\right){\left(\frac{MO}{MO_{0}}\right)}^{z_{ic}}$$

(48) $$\kappa_{2}(\xi )=4.933\xi^{2}-0.0846\xi +0.0003$$

Here, *C*_17_ = 0.007, and the threshold pit coalescence rate is *C*_18_ = *κ*_2_(*ξ*)(*f_Eu_α_* (*ξ*) + *f_Eu_β_* (*ξ*))exp[*α_a_*(*ξ*)*nF_R_*/(*RT*)][(*E_Eu_α_* (*ξ*) − *E_α_*(*ξ*))*f_Eu_α_* (*ξ*) + (*E_β_* (*ξ*) − *E_a_*(*ξ*))*f_β_* (*ξ*)]*GS*(*ξ*).

### 3.6. Calibration of Internal State Variable (ISV) Corrosion Equations with Experimental Data

**Figure 4:**
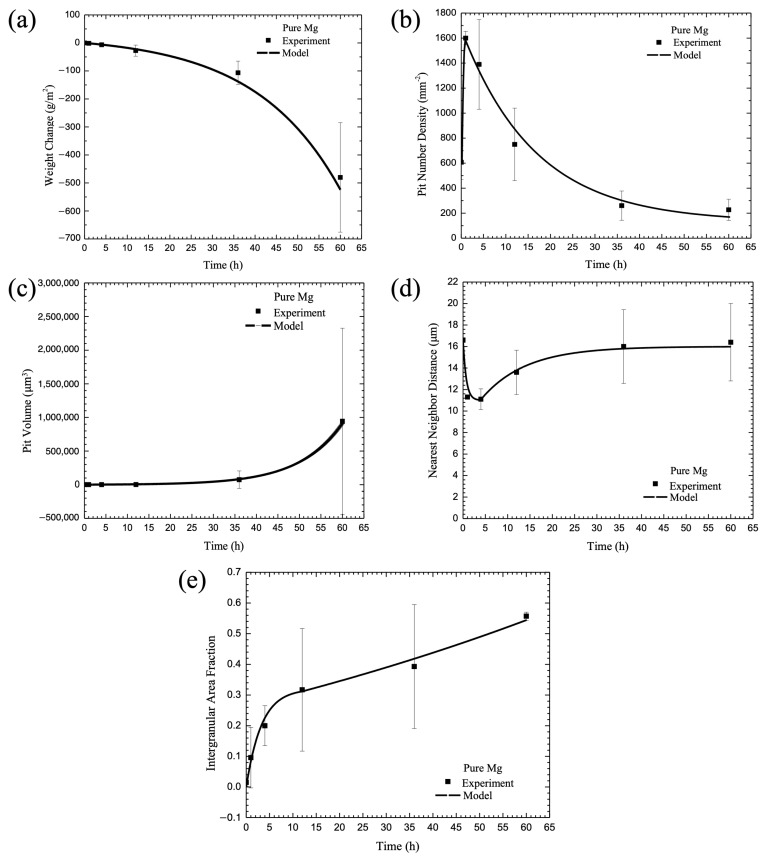
Comparison between the proposed theoretical damage framework and the corrosion experimental data of pure Mg (3.5wt.% NaCl immersion environment). The figure shows experimental data versus the model for (**a**) change in mass, (**b**) pit number density, (**c**) pit volume, (**d**) pit nearest neighbor distance, and (**e**) intergranular corrosion area fraction. The error bars show one standard deviation in each direction.

**Figure 5:**
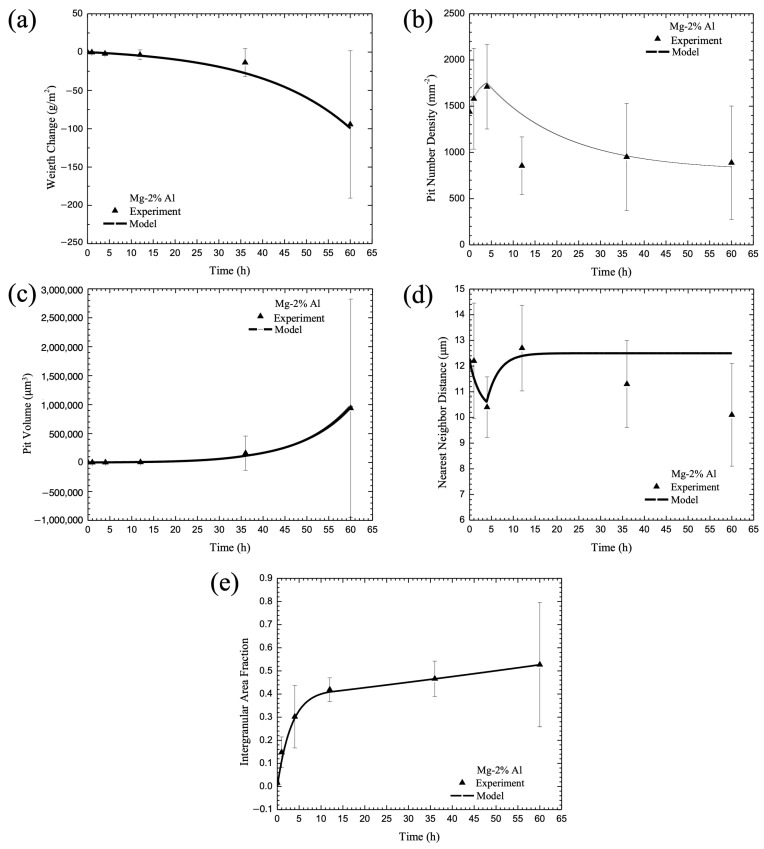
Comparison between the proposed theoretical damage framework and the corrosion experimental data of Mg-2% Al alloy (3.5wt.% NaCl immersion environment). The figure shows experimental data versus the model for (**a**) change in mass, (**b**) pit number density, (**c**) pit volume, (**d**) pit nearest neighbor distance, and (**e**) intergranular corrosion area fraction. The error bars show one standard deviation in each direction.

**Figure 6:**
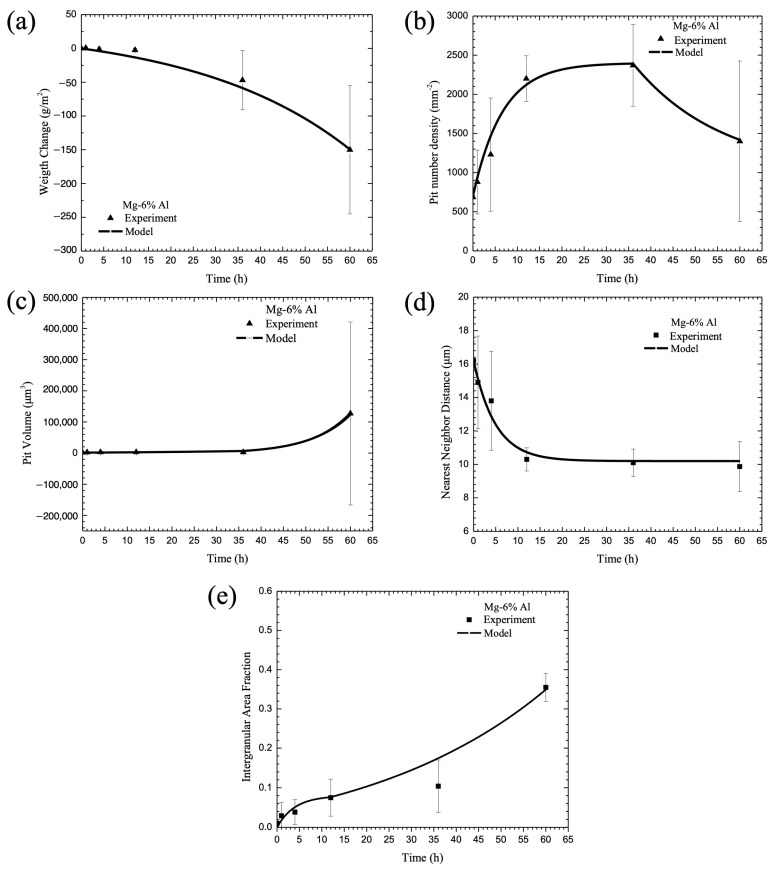
Comparison between the proposed theoretical damage framework and the corrosion experimental data of Mg-6% Al (3.5wt.% NaCl immersion environment). The figure shows experimental data versus the model for (**a**) change in mass, (**b**) pit number density, (**c**) pit volume, (**d**) pit nearest neighbor distance, and (**e**) intergranular corrosion area fraction. The error bars show one standard deviation in each direction.

The experimental data described earlier was used to determine the parameters for Equations (24)–(48), where just the chemistry percent volume fractions were the only inputs into the equations. The microstructural features, such as the particle size, particle number density, nearest neighbor distance, aluminum content in each phase, grain size, and second phase area fraction, were quantified for the cast pure Mg, Mg-2% Al, and Mg-6% Al alloys in Equation (24). The electrochemical resistance data were used to determine Equations (25)–(28) for the general corrosion rate. Then, the microstructural characteristics and electrochemical potential of each phase were included in the multiscale ISV corrosion model, and the predicted corrosion process for pitting (Equations (29)–(35)) and intergranular corrosion (Equations (36)–(38)) were compared with the experimental data, as shown in Figure 4, Figure 5 and Figure 6. Good agreement was found between the model and the experimental data. We chose model constants set with the lowest error between the model curve and the experimental data by performing least-squares optimization. Note that the calibrated model constants and used parameters are summarized in Table 3 and Table 4.

Figure 4, Figure 5 and Figure 6 show data collected for pure Mg, Mg-2 wt% Al, and Mg-6 wt% Al, respectively. Each of the figures show the (a) change in mass, (b) pit number density, (c) pit area fraction, (d) pit nearest neighbor distance, and (e) intergranular corrosion area fraction for both data collected over 60 h and the model fit. When looking at Figure 4, one can see that the ISV model followed the behavior of each of the experimental values well, following the data points or falling within the standard deviation lines for every point. In Figure 5, the ISV model followed the data relatively well, although less perfectly than the pure Mg. Between all five graphs, there were three data points that did not fall on the predictive model line, with one occurring in the pit number density, and two on the nearest neighbor distance. For all three points, they fell below the model line. For Figure 6, again, the ISV model followed the data well, with only one point not following on the line for the intergranular area fraction; as with Figure 5, it fell below the ISV model line. 

## 4. Discussion

Some interesting observations were made about the mechanism changes that occurred as the amount of aluminum increased in the magnesium base material. When considering general corrosion at 60 h, the mass loss was the greatest for the pure magnesium, where it lost approximately 500 g/cm^2^. With an increase in aluminum, the loss nonlinearly decreased when compared with the percent aluminum included. For 2% aluminum, the mass loss was approximately 150 g/cm^2^, and for 6% aluminum, the mass loss was approximately 100 g/cm^2^. This weight loss difference makes sense, as the addition of aluminum can help to produce a protective oxide film, which is harder to degrade than magnesium oxide, thereby reducing the corrosion rate of the magnesium and decreasing the mass loss. For the purpose of the ISV model, it is clear that the higher the amount of aluminum, the more the general corrosion rate was inhibited. This effect was clearly captured in the ISV model and shown in Figure 4a, Figure 5a and Figure 6a, where the ISV model followed the data points almost exactly. 

Although the corrosion rate decreased with increasing aluminum, the pitting rate increased. While the trend was similar, where the pit number density first increased, followed by a decrease when the pits coalesced, overall, the time was greatly different. When examining the percentage of aluminum in the magnesium for pit nucleation, the time when coalescence took over increased as the amount of aluminum increased. For the pure magnesium material, the rate of pitting switched to coalescence within the first hour of starting the test, as shown in Figure 4b. For the 2% aluminum material, the time increased to 4 h, as shown in Figure 5b, and for the 6% aluminum material, the time increased to 36 h, as shown in Figure 6b. In addition, the number of pits present when coalescence began increased as the aluminum percentage increased. For pure Mg, the number of pits was 1800/mm^2^, for Mg-2 wt% Al, it was 1850/mm^2^, and for Mg-6 wt% Al, it was 2300/mm^2^. Basically, with more aluminum added into the magnesium base, one garnered more corrosion nucleation sites for three reasons: (1) the general corrosion rate decreased with the addition of more second-phase material, thus allowing for pitting to take over; (2) the grain size decreased, making more surface area for the eutectic region where the particles are dominant, and thus, giving more galvanic activity; and (3) the second-phase particles were larger, thus creating a greater opportunity for corrosion nucleation to occur. While there were more pits present as the percentage of aluminum increased, the time to coalescence took longer as the percentage of aluminum increased. This was due to the fact that the pits were further apart at the time of nucleation (Figure 4d, Figure 5d and Figure 6d). If the pits are farther apart, then it would take a longer period of time for the pits to interact and begin to coalesce. When we looked only at the pit coalescence (Figure 4d, Figure 5d and Figure 6d), the trend was the inverse of the pit nucleation trends. Literally, at the same times that the pit nucleation switched from a positive increase to a negative decrease, the coalescence data trends inversely changed. This would indicate that when the pits approached each other, decreasing the nearest neighbor distance, they were about to coalesce with close neighbors. At the point of coalescence, there were now fewer pits, which decreased the pit number density (Figure 4b, Figure 5b and Figure 6b) and increased the nearest neighbor distance (Figure 4d, Figure 5d and Figure 6d).

The combined behavior of pit nucleation and pit coalescence seemingly contradicted the growth rate (Figure 4c, Figure 5c and Figure 6c), where the pit growth rate was basically the same regardless of aluminum percentage. This could be attributed to the volume of the pit, which barely changed between t_0_ and t_35_, before increasing to t_60_. However, this lack of volume change could be attributed to two values not seen in the volume graph, specifically the weight loss from general corrosion and the pit area. With the weight loss from general corrosion, the top surface would be removed, reducing the overall volume. However, as the pit grows in size, as seen in the decrease in pit number density, the area would increase, increasing the overall volume. With the competing corrosion mechanisms—general corrosion removing and the area of the pit growing—the volume appeared to hold steady. Overall, when looking at the pit growth individually, this was the slowest mechanism of the mechanisms observed; however, when looking at it as a combined behavior of other mechanisms, one can see that its behavior would be affected by what occurred on the surface. The sharp increase at t_60_ occurred because the pit coalescence that occurred then increased the volume of individual pits far faster than the interaction between the general corrosion that removed the surface and the pit area that increased across the surface.

Intergranular corrosion operated in a power law form as a function of time, and as the percentage of aluminum increased, the magnitude of the corroded area decreased (Figure 4e, Figure 5e and Figure 6e). This change in the intergranular corrosion makes sense because the presence of aluminum would increase the protective passive film, especially along the β phase, resulting in more difficulty corroding along the grains. 

Overall, one can see that the Mg-6 wt% Al incurred less intergranular corrosion and general corrosion but greater pitting corrosion when compared with the pure magnesium, with the Mg-2 wt% Al in between the pure Mg and the 6 wt% aluminum. Both the general corrosion and intergranular corrosion would be affected by the aluminum addition, which would create a passive film that is more protective than the one created by the magnesium. This passive film would slow down the general corrosion, as the overall surface attack would need to break through the passive film before it could degrade the metal, and intergranular corrosion, as it would be difficult to corrode along the grains where the β phase is the most prevalent. However, the passive film would be less effective the farther away from the β phase, meaning that places within the α phase that are not covered by the passive film would be easy targets for pitting corrosion to begin. Once the passive film was broken through, more pits could form, although it did take longer for the pits to nucleate (Figure 6b). However, for the pits to grow and then coalesce, the pits needed to break through the passive film closer to the β phase, which resulted in a slower overall nucleation (Figure 6b) and coalescence (Figure 6d). All of these interactions between the passive film formation and weight loss, pit nucleation, pit growth, and pit coalescence, along with the passive film formation and intergranular corrosion, are incredibly complex. However, with the additions to the ISV model provided above, it is clear that the new model accurately follows the corrosion behavior. This new model can account for the changes in behavior based on the addition of a passivating metal in different weight percentages.

## 5. Conclusions

A multiscale corrosion constitutive model of Walton et al. (2014) [21] was updated with a more physical multiscale basis with calibrated results coming only from the initial volume fractions of the two elements: magnesium and aluminum. The kinetics ISV equations incorporated the Butler–Volmer equation and was microstructure-dependent in order to distinguish between the different corrosion damage mechanisms: general corrosion, pitting corrosion, and intergranular corrosion. This new model provided for an accurate prediction of the various aspects of corrosion, regardless of the weight percentage of aluminum added. The theoretical framework of this model can be implemented as a finite element code, XFEM code, peridynamics code, or phase field code for engineering analysis and can be employed for other material systems. The long-term goal is to just pick elements of the periodic table and their associated weight percentages and then be able to predict the corrosion rates. This is what was accomplished herein for magnesium and aluminum.

## Figures and Tables

**Table 1:** Microstructural parameters for the cast magnesium alloys.

Parameters	Pure Mg	Mg-2% Al	Mg-6% Al	Units
*GS*	1000	500	161	mm
$D_{Eu\_\alpha }$	0	9.79	22	mm
$\delta_{Eu\_\alpha }$	0	120	410	mm^−2^
$NND_{Eu\_\alpha }$	0	36	31.43	mm
$f_{Eu\_\alpha }$	0	0.3363	0.20	-
$P_{Eu\_\alpha }$	0	0.03	0.10	-
$P_{matrix}$	0	0.015	0.03	-
$D_{\beta }$	0	2.49	5.2	mm
$\delta_{\beta }$	0	769	1434	mm^−2^
$NND_{\beta }$	0	0	13.172	mm
$f_{\beta }$	0	0.001	0.049	-

**Table 2:** Electrochemical potential for the Mg-Al solution phases and polarization resistance of the pure Mg in 3.5 wt.% NaCl solution.

Parameters	Values	Units
$E_{Mg}$	−1.67	Volts
$E_{alpha\_1.5Al}$	−1.63	Volts
$E_{alpha\_3Al}$	−1.61	Volts
$E_{alpha\_4Al}$	−1.59	Volts
$E_{alpha\_5Al}$	−1.57	Volts
$E_{alpha\_9Al}$	−1.52	Volts
$E_{\beta }$	−1.43	Volts
R_p_	137	Ohm/cm^2^

Note that all the potential values are relative to the standard calomel electrode.

**Table 3:** Physical constants used for model application.

Parameter	Values
*F* (C/mol)	96,485
*M* (g/mol)	24.305
*z*	2
*k_e_* (Nμm^2^/C^2^)	8.987 × 10^21^
*q_1_*	1 × 10^−19^
*q_2_*	1 × 10^−19^
$\varepsilon_{0}$ (C^2^/Nμm^2^)	8.85419 × 10^−24^
*MO/MO*_0_	1
*Z_ic_*	1

**Table 4:** Material parameter values used for model calibration.

Parameter	Mg-0%Al	Mg-2%Al	Mg-6%Al
C1	−250	−157	−190
C2	85	40	45
C3	2.5	0.5	0.15
C4	1650	180	2400
C5	0.06	0.05	0.05
C6	130	800	1000
C7	0.04473	0.075	0.3
C8	1585	950	400
C9	0	0	0.08
C10	0	0	40
C11	1.3	0.4	0.2
C12	11	10.2	10.2
C13	0.1	0.35	0
C14	16	12.5	0
C15	0.3	0.3	0.25
C16	0.32	0.42	0.08
C17	0.007	0.003	0.024
C18	0.27	0.35	0.05

## Data Availability

The original contributions presented in the study are included in the article, further inquiries can be directed to the corresponding authors.

## References

[1] Gordon B.M. (2013). Corrosion and Corrosion Control in Light Water Reactors. JOM.

[2] Makar G.L., Kruger J. (1993). Corrosion of magnesium. Int. Mater. Rev..

[3] Perez T.E. (2013). Corrosion in the Oil and Gas Industry: An Increasing Challenge for Materials. JOM.

[4] Aghion E., Bronfin B. (2000). Magnesium Alloys Development towards the 21st Century. Mater. Sci. Forum.

[5] Friedrich H., Schumann S. (2001). Research for a “new age of magnesium” in the automotive industry. J. Mater. Process. Technol..

[6] Froats A., Aune T.K., Hawke D., Unsworth W., Hillis J. (1987). Corrosion of magnesium and magnesium alloys. ASM Handbook.

[7] Cho H.E., Hammi Y., Francis D.K., Stone T., Mao Y., Sullivan K., Wilbanks J., Zelinka R., Horstemeyer M.F. (2018). Mi-crostructure-sensitive, history-dependent internal state variable plasticity-damage model for a sequential tubing process. Integrated Computational Materials Engineering (ICME) for Metals: Concepts and Case Studies.

[8] Cho H., Hammi Y., Bowman A., Karato S.-I., Baumgardner J., Horstemeyer M. (2019). A unified static and dynamic recrystallization Internal State Variable (ISV) constitutive model coupled with grain size evolution for metals and mineral aggregates. Int. J. Plast..

[9] Horstemeyer M.F., Bammann D.J. (2010). Historical review of internal state variable theory for inelasticity. Int. J. Plast..

[10] Coleman B.D., Gurtin M.E. (1967). Thermodynamics with Internal State Variables. J. Chem. Phys..

[11] Gurson A.L. (1977). Continuum Theory of Ductile Rupture by Void Nucleation and Growth: Part I—Yield Criteria and Flow Rules for Porous Ductile Media. J. Eng. Mater. Technol..

[12] Bammann D.J., Chiesa M.L., Horstemeyer M.F., Weingarten L.I. (1993). Failure in ductile materials using finite element methods. Struct. Crashworthiness Fail..

[13] Cocks A.C.F., Ashby M.F. (1980). Intergranular fracture during power-law creep under multiaxial stresses. Met. Sci..

[14] Horstemeyer M.F., Gokhale A.M. (1999). A void–crack nucleation model for ductile metals. Int. J. Solids Struct..

[15] Horstemeyer M.F., Lathrop J., Gokhale A., Dighe M. (2000). Modeling stress state dependent damage evolution in a cast Al–Si–Mg aluminum alloy. Theor. Appl. Fract. Mech..

[16] Chandler M.Q., Bammann D.J., Horstemeyer M.F. (2013). A Continuum Model for Hydrogen-Assisted Void Nucleation in Ductile Materials. Model. Simul. Mater. Sci. Eng..

[17] Peterson L.A., Horstemeyer M.F., Lacy T.E., Moser R.D. (2020). Experimental Characterization and Constitutive Modeling of an Aluminum 7085-T711 Alloy Under Large Deformations at Varying Strain Rates, Stress States, and Temperatures. Mech. Mater..

[18] Cho H., Zbib H.M., Horstemeyer M.F. (2022). An Internal State Variable Elastoviscoplasticity-Damage Model for Irradiated Metals. J. Eng. Mater. Technol..

[19] Dimitrov N., Liu Y., Horstemeyer M.F. (2020). An electroplastic internal state variable (ISV) model for nonferromagnetic ductile metals. Mech. Adv. Mater. Struct..

[20] Malki M., Horstemeyer M.F., Cho H.E., Peterson L.A., Dickel D., Capolungo L., Baskes M.I. (2024). A Multiphysics Thermoelastoviscoplastic Damage Internal State Variable Constitutive Model including Magnetism. Materials.

[21] Walton C.A., Horstemeyer M., Martin H.J., Francis D. (2014). Formulation of a macroscale corrosion damage internal state variable model. Int. J. Solids Struct..

[22] Amiri M., Arcari A., Airoldi L., Naderi M., Iyyer N. (2015). A continuum damage mechanics model for pit-to-crack transition in AA2024-T3. Corros. Sci..

[23] Chen Z., Bobaru F. (2015). Peridynamic modeling of pitting corrosion damage. J. Mech. Phys. Solids.

[24] Jafarzadeh S., Chen Z., Li S., Bobaru F. (2019). A peridynamic mechano-chemical damage model for stress-assisted corrosion. Electrochimica Acta.

[25] Xia D., Deng C., Chen Z., Li T., Hu W. (2022). Modeling localized corrosion propagation of metallic materials by peri-dynamics: Progresses and challenges. Acta Metall. Sin..

[26] Cui C., Ma R., Martínez-Pañeda E. (2020). A phase field formulation for dissolution-driven stress corrosion cracking. J. Mech. Phys. Solids.

[27] Mai W., Soghrati S., Buchheit R.G. (2016). A phase field model for simulating the pitting corrosion. Corros. Sci..

[28] Mai W., Soghrati S. (2017). A phase field model for simulating the stress corrosion cracking initiated from pits. Corros. Sci..

[29] Hu P., Meng Q., Hu W., Shen F., Zhan Z., Sun L. (2016). A continuum damage mechanics approach coupled with an improved pit evolution model for the corrosion fatigue of aluminum alloy. Corros. Sci..

[30] Lemaitre J., Chaboche J.-L. (1990). Mechanics of Solid Materials.

[31] Li A., Hu W., Zhan Z., Meng Q. (2022). A novel continuum damage mechanics-based approach for thermal corrosion fatigue (TCF) life prediction of aluminum alloys. Int. J. Fatigue.

[32] Li X., Zhao Y., Qi W., Wang J., Xie J., Wang H., Chang L., Liu B., Zeng G., Gao Q. (2019). Modeling of Pitting Corrosion Damage Based on Electrochemical and Statistical Methods. J. Electrochem. Soc..

[33] Gutman E.M. (1998). Mechanochemistry of Materials.

[34] Valor A., Caleyo F., Alfonso L., Rivas D., Hallen J. (2007). Stochastic modeling of pitting corrosion: A new model for initiation and growth of multiple corrosion pits. Corros. Sci..

[35] Wei R.P. (2002). Environmental considerations for fatigue cracking. Fatigue Fract. Eng. Mater. Struct..

[36] Liu C., Kelly R.G. (2019). A review of the application of finite element method (FEM) to localized corrosion modeling. Corrosion.

[37] Bailly-Salins L., Borrel L., Jiang W., Spencer B.W., Shirvan K., Couet A. (2021). Modeling of high-temperature corrosion of zirconium alloys using the extended finite element method (X-FEM). Corros. Sci..

[38] Jin H., Yu S. (2022). Study on corrosion-induced cracks for the concrete with transverse cracks using an improved CDM-XFEM. Constr. Build. Mater..

[39] Zhang X., Okodi A., Tan L., Leung J., Adeeb S. (2020). Failure Pressure Prediction of Cracks in Corrosion Defects Using XFEM. International Pipeline Conference.

[40] Guo S., Wang H., Han E.H. (2024). Cellular Automata Simulation of Pitting Corrosion of Metals: A Review. Corrosion Modelling with Cellular Automata.

[41] Reinoso-Burrows J.C., Toro N., Cortés-Carmona M., Pineda F., Henriquez M., Madrid F.M.G. (2023). Cellular Automata Modeling as a Tool in Corrosion Management. Materials.

[42] Zhi Y., Jiang Y., Ke D., Hu X., Liu X. (2024). Review on Cellular Automata for Microstructure Simulation of Metallic Materials. Materials.

[43] Horstemeyer M.F. (2012). Integrated Computational Materials Engineering (ICME) for Metals: Reinvigorating Engineering Design with Science.

[44] Horstemeyer M.F. (2018). Integrated Computational Materials Engineering (ICME) for Metals: Concepts and Case Studies.

[45] Butler J.A.V. (1924). Studies in heterogeneous equilibria. Part II.—The kinetic interpretation of the nernst theory of electromotive force. Trans. Faraday Soc..

[46] Butler J.A.V. (1932). The thermodynamics of the surfaces of solutions. Proc. R. Soc. Lond. Ser. A Contain. Pap. A Math. Phys. Character.

[47] Butler J.A.V. (1936). Hydrogen overvoltage and the reversible hydrogen electrode. Proc. R. Soc. Lond. Ser. A. Math. Phys. Sci..

[48] Erdey-Gruz T., Volmer M. (1930). The theory of hydrogen overvoltage. Z. Phys. Chem.

[49] Gurtin M.E., Drugan W.J. (1981). An Introduction to Continuum Mechanics. J. Appl. Mech..

[50] Martin H.J., Horstemeyer M.F., Wang P.T. (2011). Structure–property quantification of corrosion pitting under immersion and salt-spray environments on an extruded AZ61 magnesium alloy. Corros. Sci..

[51] Martin H.J., Horstemeyer M.F., Wang P.T. (2010). Effects of Variations in Salt-Spray Conditions on the Corrosion Mechanisms of an AE44 Magnesium Alloy. Int. J. Corros..

[52] Walton C.A., Martin H.J., Horstemeyer M.F., Wang P.T. (2012). Quantification of corrosion mechanisms under immersion and salt-spray environments on an extruded AZ31 magnesium alloy. Corros. Sci..

[53] Liu M., Uggowitzer P.J., Nagasekhar A., Schmutz P., Easton M., Song G.-L., Atrens A. (2009). Calculated phase diagrams and the corrosion of die-cast Mg–Al alloys. Corros. Sci..

[54] (2007). Standard Practice for Operating Salt Spray (Fog) Apparatus.

[55] Faraday M. (1855). Experimental Researches in Electricity, Vols. I. and II. Richard and John Edward Taylor.

[56] Faraday M. (1859). Experimental Researches in Chemistry and Physics.

[57] (1987). ASM Handbook.

[58] Zhu L. (2002). Study of Corrosion Pits in Chloride Solution. Ph.D. Thesis.

[59] Ernst P., Newman R.C. (2002). Pit growth studies in stainless steel foils. I. Introduction and pit growth kinetics. Corros. Sci..

[60] Coulomb C.A. (1785). Premier mémoire sur l‘électricité et le magnétisme. Histoire de l’Académie Royale des Sciences.

[61] Coulomb C.A. (1785). Second mémoire sur l‘électricité et le magnétisme. Histoire de l’Académie Royale des Sciences.

[62] Coulomb C.A. (1785). Troisième mémoire sur l‘électricité et le magnétisme. Histoire de l’Académie Royale des Sciences.

[63] Maxwell J.C. (1878). On Stresses in Rarified Gases Arising from Inequalities of Temperature. R. Soc. Proc..

[64] Tedmon C.S., Vermilyea D.A., Rosolowski J.H. (1971). Intergranular Corrosion of Austenitic Stainless Steel. J. Electrochem. Soc..

[65] Birbilis N., Ralston K.D., Virtanen S., Fraser H.L., Davies C.H.J. (2010). Grain character influences on corrosion of ECAPed pure magnesium. Corros. Eng. Sci. Technol..

[66] Shi Z., Liu M., Atrens A. (2010). Measurement of the corrosion rate of magnesium alloys using Tafel extrapolation. Corros. Sci..

[67] Tafel J., Hahl H. (1907). Vollständige Reduktion des Benzylacetessigesters. Berichte Der Dtsch. Chem. Gesell-Schaft.

[68] Stern M., Geary A.L. (1957). Electrochemical Polarization: I A Theoretical Analysis of the Shape of Polarization Curves. J. Electrochem. Soc..

[69] Deshpande K.B. (2011). Numerical modeling of micro-galvanic corrosion. Electrochimica Acta.

